# The First Stage of Labor*Read at Hearne meeting Brazos Valley Medical Association, November 13, 1899.

**Published:** 1900-01

**Authors:** W. C. Taylor

**Affiliations:** Branchville, Texas


For Texas Medical Journal.

### The First Stage of Labor.*

BY W. C. TAYLOR, BRANCHVILLE, TEXAS.

The management of labor comprises two elements. First the careful observation of every successive stage of the process of parturition, which presupposes a familiarity with the mechanism by which nature herself affects delivery; and secondly, the application of artificial assistance at the proper time to accomplish results for which nature alone is insufficient, to obviate the disastrous results of intercurrent accidents.

In the cases first to be considered, those in which nature is adequate to every step in the progress, the former element in the management will predominate, and the attitude of the practitioner is chiefly one of expectancy; an expectancy which, however, should always be armed with a definite knowledge of nature's methods, while as we pass on through the possible abnormalities and complications of the parturient process, the second or active element enters largely into the management. The average mortality of labor, as met with in general practice, is about 1 in 120; that is, 1 in 120 women delivered at term, dies within a month of child birth. Under good obstetric management, women in comfortable circumstances in life should, it is believed, show a lower percentage of mortality. To begin with a simple case, the mother is of sufficient age to have reached her full sexual development and not so old as to have the parts involved in parturition in too rigid and unyielding a state; she

---

*Read at Hearne meeting Brazos Valley Medical Association, November 13, 1899.

has the pelvis and genital passage of sufficient capacity and has good muscular strength. The child presents by the vertex., *i. e.*, lies toward the os uteri and will be the first to impinge upon the examining finger.

The presentation of the vertex necessitates that the head shall be well flexed upon the chest. The occiput is directed toward the left acetabulum, which is first position (O L A).

In the management of a simple case of this kind the practitioner will need little in the way of apparatus, yet for the reason that he never can tell in advance what will be the character of a given labor, he should always go prepared with the following articles: An English red gum elastic catheter, size 6 to 8; needles with a long curve and needle holder, either silk or carbolized catgut or silver wire for sutures; a Davidson's syringe with long nozzle for uterine injections. A hypodermic syringe with Magendie's solution, and a solution of ergotine or of an extract of ergot, suitable for subcutaneous injection, a pair of obstetric forceps, a small bottle of F. E. of ergot. The per. chlo. of iron or tincture of iodine, tincture of opium, and a solution of chloral, carbolic acid and a solution of bichloride of mercury, chloroform or sul. ether. At the house of the patient ice, hot water, brandy and scissors should be prepared. On reaching the patient the attendant should see that the bed is properly arranged. It should be, if possible, in a light and airy room; the bed should be accessible from both sides. The mattress should be protected by a rubber sheet, placed under the lower sheet, above the latter should be a drawn sheet folded to several thicknesses and about two feet in width. The attendant should carefully wash his hands, using a nail brush, before making any examination of the patient. It is well to further disinfect the hands by a solution of carbolic acid or a bichloride solution.

During the first stage of labor the patient need not be undressed, but should wear a wrapper that can be easily removed. When she takes to bed at the beginning of the second stage or before the escape of the waters, the night dress should be gathered up above the waist in order to preserve it from the discharges. As soon as possible after arriving the attendant should make an examination, at first of the abdomen (the patient lying on her back), both by inspection and palpation, and then digitally (per vaginum.) If he arrives soon after the onset of pains, he will find that the os externum is just beginning to dilate, but will not yet admit the finger. The inner os is also begining to yield or the canal of the cervix may already be taken into the uterine cavity through the unperceived uterine contractions of a few days preceding the onset of pains, which clinically marks the beginning of labor.

If a diagnosis of normal presentative can be made out at the first examination and if the pains are effective, the accoucheur has evidence that all is beginning well and may postpone further examination. If the bowels and bladder have not been emptied for some time, this may now be done by injection of cold water in the one case and by the catheter, if necessary, in the other. This should always be done if the pains of the first stage are troublesome but do not appear to accomplish much toward the dilation of the os. The pains, if true ones, are sharp, cutting in character, recur periodically, with gradually increasing frequency, and under them the inner os yields and the cervix gets thinner and gradually opens until the protecting membranes are felt swelling down upon the examining finger at each recurring pain. The examination should generally be begun between the pains, but it is usually desirable to continue it during the pains. A copius glairy secretion, having a lubricating function has been pouring out into the vagina and vulva. The dilatation of the os is affected most easily by the bag of waters in front of the child's head, which acts as an elastic wedge. Hence, as a rule, this bag should not be broken artificially until its mission, the dilatation of the os, be complete, when, however, there is an excessive amount of the amniotic fluid so that the uterus is over distended and its contractile power therefore weakened, it is necessary to rupture the membranes artificially. This may easily be done by the use of a straightened hair pin carried in the groove between the index and middle finger and held against the projecting membranes during a pain.

The practitioner sometimes feels an uncertainty in regard to whether he should remain by his patient or leave her during the first stage. It is not customary for the accoucheur to remain in continuous attendance through the whole labor unless specially requested to do so and paid accordingly.

After having watched the progress for a time and satisfied himself that all is well, he may go away for a time, leaving word where he may be found.

IRREGULAR PAINS.

In the first stage of labor pains are frequently defective by reason of their short duration. As a rule, short, cramp-like pains occasion acute suffering, when they recur with little or no intermission between, they are very exhausting to the patient, as the cervix in such cases is tense and rigid. It is to this condition that the delay is attributed. If, however, the tissues of the cervix are healthy the presentation is normal and the pains preserve their expulsive character, rigidity of the cervix is never an obstacle to delivery. The activity of the organic changes which tend to softening and dilatation is closely related to the activity of the uterine contractions.

The uterine contractions may be abnormal from the commencement of labor; more frequently the loss of their expulsive character is a secondary condition.

In many primiparous women, labor progresses in an auspicious manner for a time, inspiring hopes of a speedy termination. Then the cervix which had been dilating favorably becomes rigid, the sufferings of the patient during each contraction are enhanced, and further advance is arrested.

### TREATMENT OF PROTRACTED FIRST STAGE.

The treatment of protracted first stage has for its object the mitigation of pain, and the restoration of their expulsive character to the uterine contractions. No plan of action should be decided upon without first investigating the cause of delay. The suspensive influence of a full bladder or rectum is always to be borne in mind. Breech and shoulder presentations, and a contracted pelvis are objects that should be looked after. A faulty position of the uterus should, if possible, be rectified by suitable abdominal supports. Adhesions of the membranes to the lower uterine segment should be dissected up by the index finger. In hydramnion rupture of the membranes is sometimes serviceable.

If the length of the labor is simply due to insufficient uterine action, the conduct of the accoucheur will depend upon the frequency and severity of the pains, and the endurance of the patient. If they occur at such intervals and with such mildness that the patient can eat, sleep, and attend to ordinary household duties, the dilatory progress should cause no alarm. In pathological conditions it is the element of pain that is most to be dreaded. Pain, long continued, is a powerful nerve depressant, when combined with starvation and loss of sleep. It greatly impairs a woman's capacity to resist the perils of the puerperal period.

While the indications for treatment are clear, it is not easy in every case to decide whether the remedy should be applied, first, to the relief of pain, or whether efforts should be directed to the acceleration of labor so as to speedily place the patient beyond the hazards of parturition. As a rule anodynes are appropriate in cases where the cervix is but slightly dilated, while accelerative measures are preferable in cases where the first stage is already advanced.

The most appropriate pain-stilling agents are, the warm bath, chloral, either by the stomach or by rectal injections, chloroform and morphine. It is usually convenient to begin with chloroform and then sustain its action with morphine and the rectal administration of chloral, suspending the chloroform as soon as the effect of the latter is developed. Anodynes often accomplish wonders in one of two ways; when, owing to the prolongation of the labor and its attendant pain, the patient's nervous energies have become exhausted and the arrest of pain enables the woman to sleep, and with the recuperation of power that comes upon awakening, good pains follow which bring the labor to a speedy termination. In other cases, after the employment of anodynes, the parts relax and an acceleration of labor follows.

If pain-stilling agents do no good, or if the first stage of labor is already far advanced, the physician should seek to restore to the pains their expulsive character to hasten delivery. The warm vaginal douche is of reputed service in cases of uterine insufficiency. Barnes' dilating bags, the introduction of a bougie into the uterus where the membranes are intact, and where the pains are weak without being cramp-like in character. The internal administration of quinine in large doses, ergot, borax and digitalis are sometimes beneficial. The vaginal douche has a wide range of utility. Of all the resources at our command the water bags of Dr. Barnes stand at the head. Passed into the cervix and distended so as to place the canal moderately upon the stretch, they not only serve to dilate the os, but are most efficient reflex exciters of labor pains. If left in situ until expelled into the vagina by the bearing down efforts they awaken, the cervix will be found to have lost its rigidity. If necessary a larger bag can be placed the same way. An attempt to dilate the cervix rapidly and with violence is neither safe nor profitable.

